# Influence of Polymer Matrix Selection on the Properties of PVC- and PLA-Based Polymer Inclusion Membranes Containing D2EHPA

**DOI:** 10.1021/acsomega.6c04900

**Published:** 2026-08-10

**Authors:** Obed Ricardo Madrid Zayas, José Carmelo Encinas Encinas, Dora Evelia Rodríguez Félix, Jesús Manuel Quiroz Castillo, Silvia Elena Burruel-Ibarra, Benjamín Valdez-Salas, Guillermo del Carmen Tiburcio Munive, Hisila del Carmen Santacruz Ortega, Ramón Alfonso Moreno Corral

**Affiliations:** † Departamento de Investigación en Polímeros y Materiales, Universidad de Sonora, Rosales y Encinas s/n, Col., Centro, CP 83000 Hermosillo, Sonora, México; ‡ Instituto de Ingeniería, Universidad Autónoma de Baja California, 21280 Mexicali, Baja California, México; § Departamento de Ingeniería Química y Metalurgia, Universidad de Sonora, Rosales y Encinas s/n, Col., Centro, CP 83000 Hermosillo, Sonora, México

## Abstract

Polymer inclusion membranes (PIMs) are promising materials for separation processes due to their tunable structure–property relationships and compositional versatility. In this work, PIMs were prepared by solvent casting using di­(2-ethylhexyl) phosphoric acid (D2EHPA) as a carrier and either poly­(vinyl chloride) (PVC) or poly­(lactic acid) (PLA) as supporting matrices. The membranes were comparatively characterized to assess the influence of polymer selection on their physicochemical properties. FTIR analysis confirmed the successful incorporation of D2EHPA into both polymer matrices while preserving their chemical structures, while SEM–EDS revealed continuous membranes with distinct surface morphologies and average thicknesses of approximately 157 μm (PVC) and 141 μm (PLA), without evidence of macroscopic phase separation. Thermal analysis revealed matrix-dependent thermal stability and degradation behavior, with T5% values of approximately 220 °C for PVC-based membranes and 210 °C for PLA-based membranes and glass transition temperatures of approximately 60 and 58 °C, respectively. Water contact angle measurements revealed moderately wettable surfaces, with contact angles of 59.2° for PVC-based membranes and 64.3° for PLA-based membranes. Mechanical testing revealed a marked contrast: PVC-based membranes exhibited greater ductility (160.1% elongation at break) and lower stiffness (31.8 MPa), whereas PLA-based membranes showed substantially higher stiffness (369.1 MPa) and lower elongation at break (41.87%). Overall, these findings demonstrate that polymer matrix selection governs the physicochemical properties of D2EHPA-based polymer inclusion membranes, with PLA representing a promising biodegradable alternative for applications requiring enhanced stiffness and dimensional stability.

## Graphical abstract

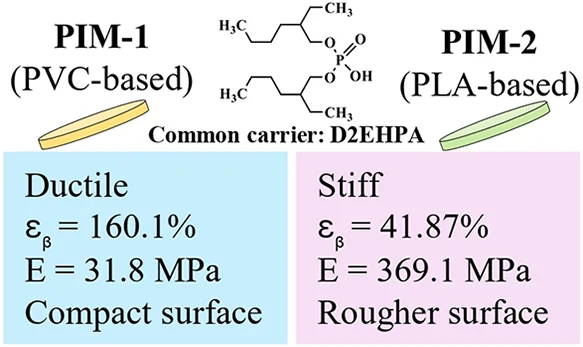

## 1 Introduction

Polymer inclusion membranes (PIMs) have attracted considerable attention as sustainable alternatives to conventional liquid–liquid extraction systems for the selective recovery of metal ions and the removal of organic pollutants such as dyes and pharmaceuticals from aqueous media.^1–6^ Their relevance arises from the integration of extraction and separation processes within a single solid phase, combining the selectivity of liquid membrane systems with the operational advantages of polymeric films, such as reduced solvent consumption and improved handling stability.^7,8^ Compared to traditional solvent extraction, PIMs have been reported to offer enhanced operational simplicity and lower environmental impact, making them promising candidates for applications in resource recovery and wastewater treatment.^4,5,9^

PIMs are typically composed of three main components: a polymer matrix, a carrier (extractant), and, in some formulations, an external plasticizer. The carrier governs the selective interaction and transport of target species across the membrane, while the polymer matrix provides the structural framework that determines mechanical integrity, chemical resistance, and long-term durability.^10,11^ The interplay between these components defines the overall membrane performance through structure–property relationships, which are strongly influenced by interactions between the carrier and polymer chains.^8,12^

In addition to their well-established use in separation processes, polymer inclusion membranes have also emerged as versatile materials for passive sampling and analytical preconcentration. In these applications, PIMs enable the selective accumulation of analytes directly from environmental waters, providing time-averaged concentrations, matrix separation, improved detection limits, and chemical speciation while supporting the principles of green analytical chemistry. These additional applications further demonstrate the versatility and growing importance of PIM technology in both environmental monitoring and separation science.^13,14^

Among the various carriers reported in the literature, organophosphorus extractants, ammonium-based compounds, and ionic liquids have been widely investigated due to their affinity toward metal ions.^15^ In particular, di­(2-ethylhexyl) phosphoric acid (D2EHPA) has been extensively employed because of its well-established extraction chemistry and high selectivity under acidic conditions relevant to hydrometallurgical processes.^16–18^ Beyond its extractive role, D2EHPA has been reported to act as an internal plasticizer in certain membrane systems, influencing polymer chain mobility and modifying membrane flexibility, thermal behavior, and stability through noncovalent interactions.^19^ This dual functionality plays a critical role in determining the physicochemical properties of PIMs and is strongly dependent on the nature of the polymer matrix.

The polymer matrix is therefore a key factor in PIM design, with poly­(vinyl chloride) (PVC) being one of the most commonly employed materials due to its favorable film-forming properties and compatibility with a wide range of carriers.^20,21^ Additionally, PVC exhibits well-documented thermal degradation mechanisms involving dehydrochlorination and the formation of conjugated polyene structures, which can progressively compromise material integrity under thermal or operational stress.^22–24^ These degradation pathways are relevant when evaluating membrane stability, particularly under prolonged use.

In contrast, poly­(lactic acid) (PLA) has emerged as a biodegradable and renewable alternative, attracting increasing attention in the development of sustainable polymeric materials. Growing environmental concerns associated with the persistence of petroleum-based polymers and the generation of secondary microplastics have stimulated the search for biodegradable polymers for membrane fabrication. Among them, PLA has become one of the most promising candidates because it combines biodegradability, renewable origin, and physicochemical properties suitable for membrane preparation. Consequently, comparing PLA with the conventional PVC matrix is essential to determine whether biodegradable polymers can provide suitable membrane performance while reducing the environmental impact associated with petroleum-based materials.^25^ PLA is characterized by higher stiffness and modulus compared to PVC, as well as lower ductility and distinct thermal behavior, which may significantly influence membrane morphology and mechanical performance.^26–28^ Although its inherent brittleness and crystallization behavior may limit certain applications, these properties also suggest potential advantages in terms of dimensional stability and structural rigidity under continuous operation conditions.^29^

Despite the growing body of research on PIM systems, systematic studies directly comparing conventional petrochemical matrices such as PVC with biodegradable alternatives such as PLA under identical preparation and characterization conditions remain scarce.^4,5^ Most existing works have focused on optimizing carrier systems or evaluating transport performance, while comparatively fewer investigations have addressed the fundamental influence of the polymer matrix on physicochemical behavior.^20,30^ Consequently, no clear consensus has been established regarding how matrix selection governs the balance between mechanical performance, thermal stability, and interfacial characteristics in D2EHPA-based PIMs.

This limitation is particularly relevant for applications such as metal recovery and wastewater treatment, where membranes are exposed to chemically aggressive environments, mechanical stress, and long-term operating conditions. Under such circumstances, the distribution and mobility of the carrier within the polymer matrix become critical factors. It has been reported that carrier redistribution, aggregation, or migration toward the membrane surface can lead to morphological changes and loss of extractant, ultimately affecting membrane stability and performance.^19,31^ Therefore, controlling these phenomena is essential for improving membrane durability and efficiency.

PIMs are commonly prepared by solvent casting, in which the polymer and carrier are dissolved in a volatile solvent followed by controlled evaporation to form a homogeneous thin film.^12^ In many systems, the carrier can also contribute to plasticization effects, modifying polymer interactions and influencing membrane properties. Alternative preparation strategies, including solvent-free and thermally induced methods, have also been explored, highlighting the versatility of PIM fabrication.^32^ In addition, membrane-based separation technologies are increasingly recognized as sustainable approaches for environmental remediation and resource recovery.^33^

In this context, a direct and controlled comparison between petrochemical and biodegradable matrices becomes essential to elucidate the influence of polymer selection on membrane properties. Therefore, the present work provides a systematic comparative analysis of PIMs based on PVC and PLA using D2EHPA as a common carrier under identical preparation conditions. The membranes were fabricated by solvent casting and characterized using complementary spectroscopic, morphological, thermal, and mechanical techniques.^34,35^ This study is based on the hypothesis that replacing the conventional petroleum-based PVC matrix with biodegradable PLA modifies the physicochemical properties of D2EHPA-based polymer inclusion membranes. Accordingly, the distinct chemical nature of the two polymer matrices is expected to produce measurable differences in membrane morphology, thermal stability, surface wettability, and mechanical behavior. The polymer inclusion membranes developed in this study are intended as candidate materials for future membrane-based transport, extraction, separation, passive sampling, and analytical preconcentration applications involving different classes of target compounds.

## 2 Materials and Methods

### 2.1 Reagents

Poly­(vinyl chloride) (PVC) (low molecular weight) and poly­(lactic acid) (PLA) (145,000 g/mol) were sourced from NatureWorks and used as polymer matrices. Di­(2-ethylhexyl) phosphoric acid (D2EHPA, ≥97%) was used as the carrier. The organic solvents tetrahydrofuran (THF) and acetone were used for membrane preparation. All reagents were purchased from Sigma-Aldrich and used as received without further purification. The chemical structures of the different reagents are presented in Table 1.

**Table 1:**
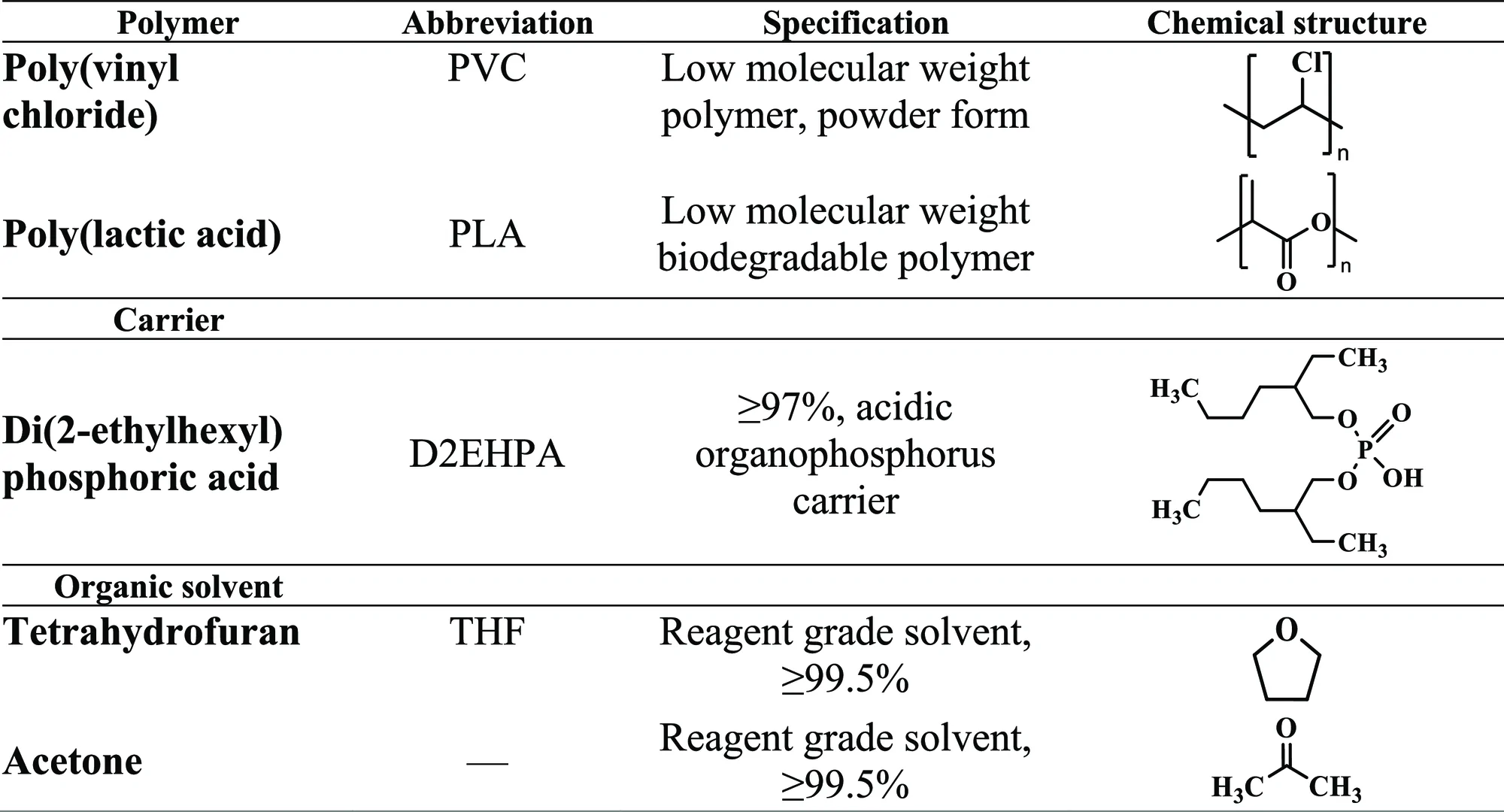
Specifications and Chemical Structures of the Different Polymers and Compounds Used for PIM Preparation

### 2.2 Methods

#### 2.2.1 Preparation of Polymeric Membranes

Solvent casting is the most widely employed technique for polymer inclusion membrane (PIM) fabrication, allowing the formation of homogeneous and self-supporting membranes after controlled solvent evaporation.^3^ In this work, PIMs were prepared using a solution casting and solvent evaporation approach at room temperature, following procedures commonly reported in the literature,^2^ with modifications depending on the polymer matrix. To prepare polymer inclusion membranes PIM-1 and PIM-2 (hereafter referred to as PIM-1 and PIM-2), appropriate amounts of each component were weighed and mixed. For PIM-1, 0.60 g of PVC was dissolved in 13.2 mL of THF under continuous magnetic stirring. After complete dissolution of the polymer, 0.60 g of D2EHPA was added. The mixture was stirred for 2 h at 120 rpm and room temperature until a homogeneous and transparent solution was obtained. The resulting solution was poured into a flat-bottom glass Petri dish (8.5 cm internal diameter), placed horizontally and loosely covered. The solvent was allowed to evaporate at room temperature for 24 h, yielding a flexible and self-supporting membrane. The resulting film was carefully peeled off and stored at room temperature until further use. PVC was used as the polymer matrix, while D2EHPA was employed as both carrier and plasticizer, consistent with its established use in metal extraction and transport through polymeric membranes.^17^

PIM-2 was prepared following the same general procedure, replacing PVC with PLA and THF with acetone, while maintaining identical amounts of polymer (0.60 g), carrier (0.60 g D2EHPA), and solvent volume (13.2 mL). The use of PLA as a supporting polymer in PIMs represents a modification of conventional systems, as biodegradable matrices in PIM fabrication remain scarcely explored. This approach enables direct comparison of the influence of the polymer matrix on membrane structure and properties.^10^ Figure 1 shows the prepared PIM-1 and PIM-2.

**Figure 1:**
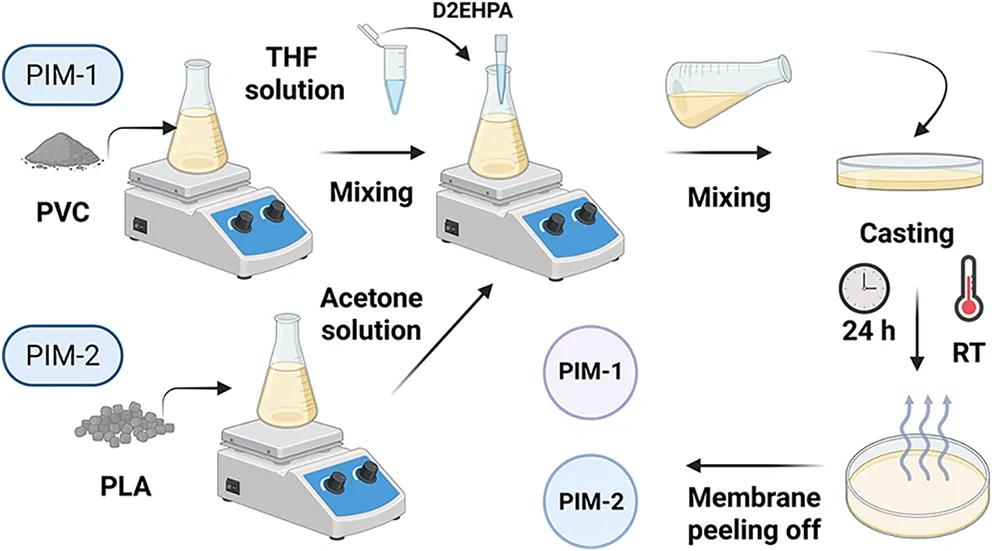
Schematic presentation of PIM fabrication. Created with BioRender.com.

### 2.3 Characterization of Membranes

As shown in Figure 2, several analytical techniques were employed to characterize the materials and assess their physicochemical properties and differences.

**Figure 2:**
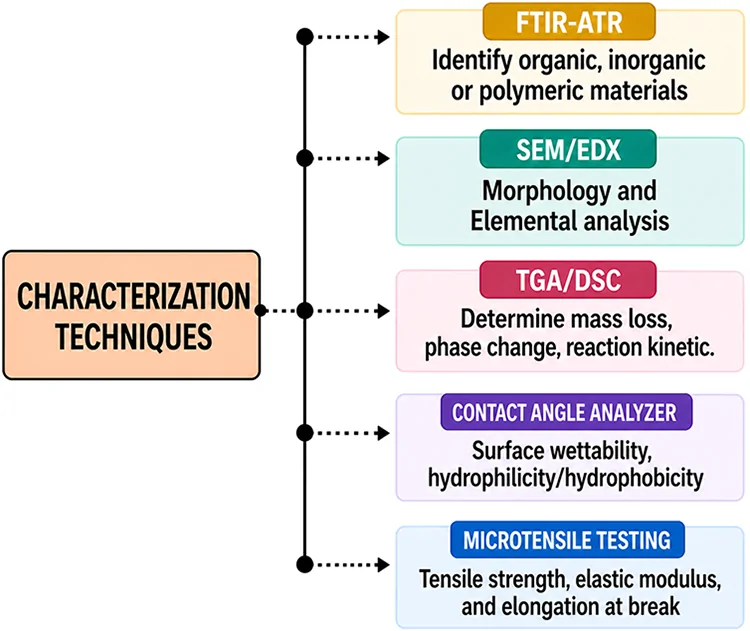
Analytical tools used to determine PIMs properties.

#### 2.3.1 Fourier-Transform Infrared (FTIR) Spectroscopy

FTIR-ATR spectra of the PIM-1 and PIM-2 membranes were recorded using the attenuated total reflectance (ATR) technique on a PerkinElmer Frontier spectrometer. Spectra were collected in the range of 4000–400 cm^–1^, with a spectral resolution of 2 cm^–1^ and 16 accumulated scans. FTIR spectroscopy is commonly employed to identify functional groups associated with polymer matrices and carriers and to evaluate potential interactions between membrane components.^36^

#### 2.3.2 Scanning Electron Microscopy (SEM) and Energy-Dispersive X-ray Spectroscopy (EDS)

The surface and cross-sectional morphology of polymer inclusion membranes was examined by scanning electron microscopy (SEM) using a JEOL JSM-5410 V microscope with an attached energy dispersive X-ray spectroscopy (EDX) analysis and elemental mapping, at an accelerating voltage of 20 kV. Samples mounted horizontally were used for surface morphology analysis, while vertically mounted cross-sectional samples were used to determine membrane thickness and internal continuity, as commonly reported for PIM systems.^10^ The membrane samples were fixed onto aluminum stubs using carbon adhesive tape. To improve electrical conductivity and reduce charging effects during analysis, the samples were coated with a thin gold layer. In addition, energy-dispersive X-ray spectroscopy (EDS) was performed on each membrane to determine their elemental composition and to confirm the incorporation and distribution of the carrier within the polymeric matrix.

#### 2.3.3 Thermogravimetric Analysis (TGA) and Differential Scanning Calorimetry (DSC)

TGA and DSC analyses were carried out using a simultaneous thermal analyzer (STA 6000, PerkinElmer). Samples (5 mg) were heated from 30 to 800 °C at a heating rate of 10 °C min^–1^ under a nitrogen atmosphere (40 mL min^–1^). Thermal analysis provides information on membrane thermal stability, degradation behavior and transitions such as glass transition temperature, which are critical for assessing membrane performance and durability.^37^

#### 2.3.4 Contact Angle Measurement

Changes in the hydrophobic character of the membrane surfaces associated with the polymer matrix content were determined from contact angle measurements. The surface contact angles of the PIMs were investigated using the sessile drop method with a contact angle analyzer (CAM-Plus, ChemInstruments). Prior to analysis, membrane samples were cut into rectangular pieces and cleaned with a dust-free air stream to remove surface contaminants. Each sample was placed on the instrument stage and carefully leveled to ensure a horizontal surface. The sessile drop technique is commonly employed for polymer membranes due to its simplicity and reproducibility for comparative wettability analysis.^3^ A 5 μL droplet of ultrapure water was deposited onto the membrane surface through a needle attached to the instrument, and the contact angle was recorded over a period of 60 s. For each membrane, ten independent measurements were performed at different surface locations, contact angle measurements were performed, and the reported values correspond to the mean ± standard deviation.

#### 2.3.5 Mechanical Properties

The mechanical properties of the membranes were measured using a TA.XT.plus texture analyzer (Texture Technologies Corp., TextureLab) equipped with tensile grips. Rectangular specimens with dimensions of 10 mm in width and 50 mm in length were tested at room temperature. The initial gauge length was fixed at 50 mm. The thickness of each membrane was measured prior to testing and used to calculate the cross-sectional area. Tensile tests were performed at a constant crosshead speed of 1 mm s^–1^. The applied force was recorded and converted to engineering stress (MPa) by normalizing with respect to the initial cross-sectional area of the sample. Strain was expressed as percentage deformation relative to the initial gauge length. Five independent samples were analyzed for each membrane formulation, and the reported values correspond to the mean ± standard deviation. From the stress–strain curves, the ultimate tensile strength (UTS), elongation at break, and apparent Young’s modulus were determined. The Young’s modulus was estimated from the slope of the initial quasi-linear region of the stress–strain curves using linear regression analysis.

## 3 Results and Discussion

### 3.1 Preparation of PIMs

The physical characteristics of the membranes prepared by the solvent casting method are summarized in Table 2. The PVC-based membranes appear homogeneous, transparent, and flexible, whereas the PLA-based membranes are homogeneous, opaque, and less flexible. These qualitative observations are consistent with the mechanical characterization, where PLA-based membranes exhibit higher stiffness.

**Table 2:** Characteristics of the PIMs Prepared with Solvent Casting Procedure

membrane	polymer matrix	carrier/plasticizer	polymer amount (g)	carrier amount (g)	organic solvent	solvent volume (mL)	membrane characteristics
PIM-1	PVC	D2EHPA	0.60	0.60	THF	13.2	Homogeneous, transparent, and flexible
PIM-2	PLA	D2EHPA	0.60	0.60	Acetone	13.2	Homogeneous, opaque, and less flexible

### 3.2 Characterization

#### 3.2.1 FTIR-ATR Spectroscopy Measurements

FTIR spectroscopy was employed to investigate the chemical structure of the membrane components and to assess potential intermolecular interactions within the polymer inclusion membranes. FTIR analysis provides valuable information about functional groups and possible interactions between the constituents of polymeric systems.^38^ In this study, FTIR spectra of PVC, PLA, D2EHPA, and the prepared membranes were analyzed and are presented in Figures 3 and 4.

**Figure 3:**
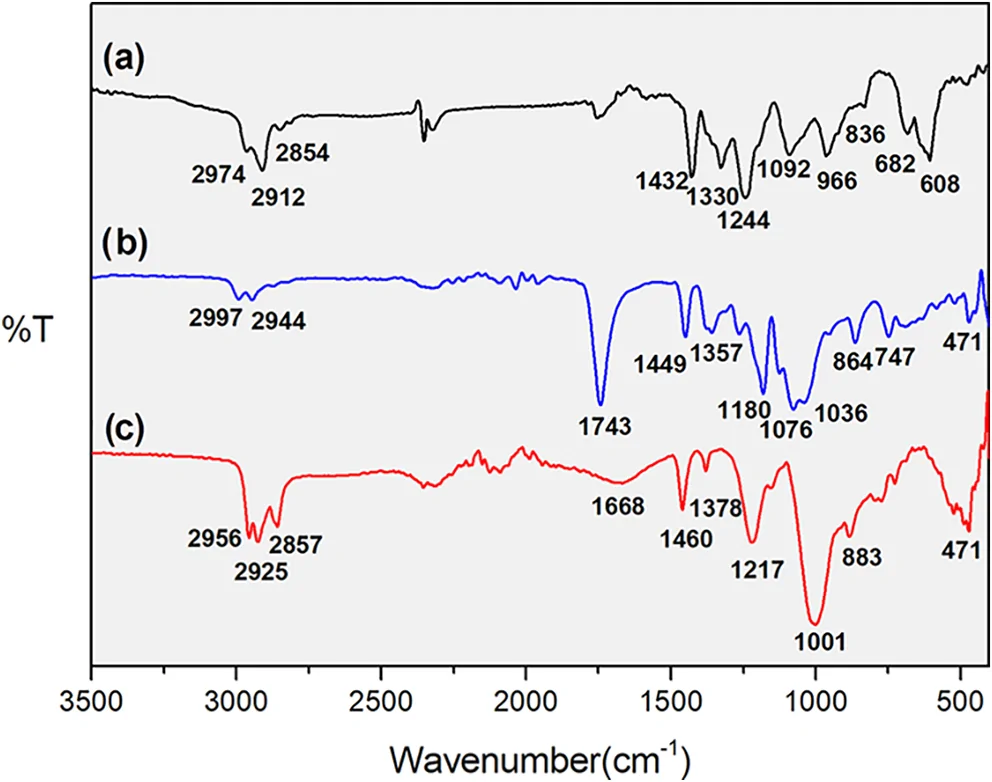
FTIR spectra of the individual components comprising the studied PIMs: PVC (a), PLA (b), and D2EHPA (c).

**Figure 4:**
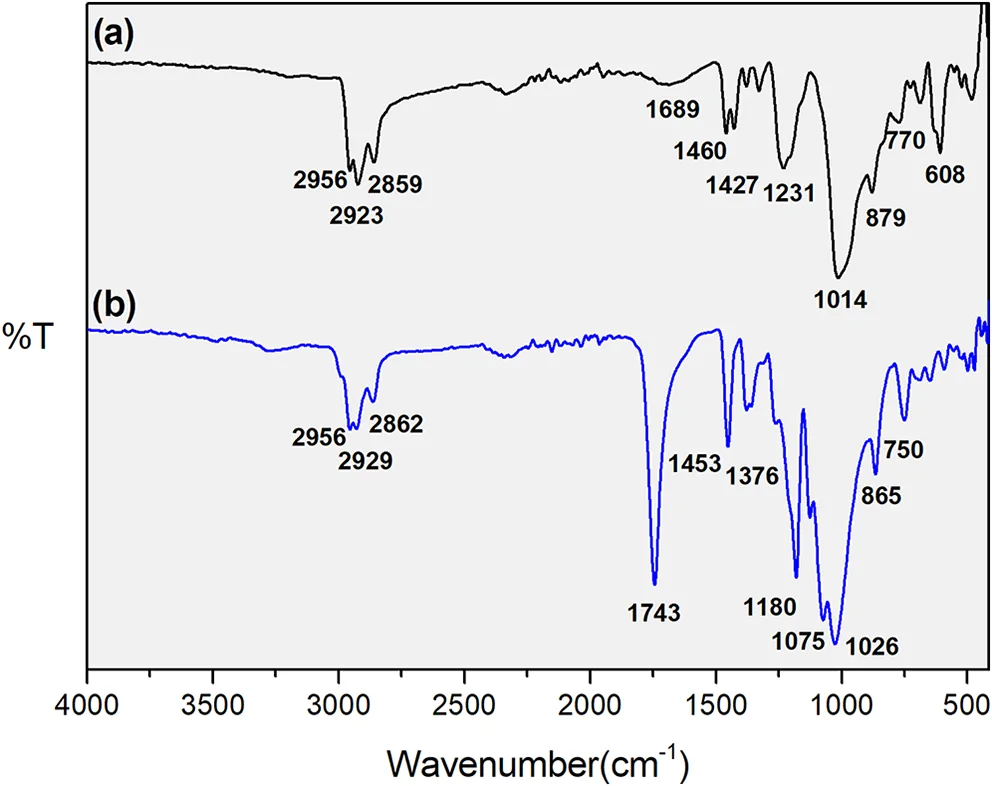
FTIR spectra of the polymer inclusion membranes (a) PIM-1 and (b) PIM-2.

The FTIR spectrum of PVC (Figure 3a) exhibits the characteristic aliphatic C–H stretching vibrations at approximately 2974 (CHCl), 2912 (CH_2_), and 2854 (CHCl) cm^–1^, together with CH_2_ bending modes at 1432 cm^–1^. The band observed around 1244 cm^–1^ can be associated with C–H wagging vibrations, while the absorption in the region between 600 and 700 cm^–1^ (notably at 610 cm^–1^) corresponds to the C–Cl stretching vibration, which is considered a fingerprint feature of PVC.^39,40^ The FTIR spectrum of PLA (Figure 3b) shows the characteristic ester carbonyl stretching band at 1743 cm^–1^, along with C–H stretching vibrations at 2944 and 2997 cm^–1^. Additional bands at 1449 and 1357 cm^–1^ are attributed to CH_3_ bending modes, while the strong absorptions observed at 1180, 1076, and 1036 cm^–1^ correspond to C–O–C stretching vibrations typical of aliphatic polyesters.^26,37^ The FTIR spectrum of D2EHPA (Figure 3c) shows characteristic features of organophosphorus compounds, including aliphatic C–H stretching vibrations in the 2956–2857 cm^–1^ region. A band at 1668 cm^–1^ is observed, which may be associated with interactions involving the phosphoryl group or overlapping contributions. Bands at 1460 and 1378 cm^–1^ correspond to CH_2_ and CH_3_ bending vibrations of the alkyl chains.^17^ The absorption at 1217 cm^–1^ is assigned to P–O stretching, while the intense band at ∼1001 cm^–1^ corresponds to P–O–C vibrations. Additional bands in the 883–471 cm^–1^ region are attributed to P–O deformation modes. These features are consistent with the structure of dialkyl phosphoric acids and their known coordination behavior.^17,18^

In the spectra of PVC-based membranes (Figure 4a), the simultaneous presence of the characteristic absorption bands of both PVC and D2EHPA confirms the successful incorporation of the carrier into the polymer matrix. The retention of the C–Cl stretching band (608 cm^–1^) and the CH_2_ bending region (1460–1427 cm^–1^), together with contributions in the 1200–1000 cm^–1^ region associated with P=O and P–O–C vibrations, indicates that both components coexist without significant structural disruption of the polymer backbone.^10,12,20,39^ Notably, slight variations in the relative intensity and subtle shifts in bands within the 1200–1000 cm^–1^ region suggest slight changes in the local chemical environment surrounding the phosphoric group. These changes are consistent with noncovalent interactions, primarily dipole–dipole interactions between the P=O group of D2EHPA and polarizable segments of the PVC chains, which may affect the spatial distribution and mobility of the carrier within the membrane.^41^ Importantly, no new absorption bands indicative of covalent bond formation were observed, confirming that the incorporation of D2EHPA into the PVC matrix occurs through physical interactions rather than chemical bonding.^10,11,20^ This behavior is consistent with the membrane formation mechanism generally reported for polymer inclusion membranes, in which the carrier is physically immobilized within the polymer matrix.^2,3,7,10^ The preservation of the characteristic functional groups indicates that membrane fabrication does not significantly alter the physicochemical integrity of the polymer or the carrier, thereby preserving the functionality required for subsequent transport and separation applications.^12,17,20^

For the PLA-based membranes (Figure 4b), the preservation of the main characteristic bands of PLA, particularly the strong carbonyl stretching band at 1743 cm^–1^, confirms that the polymer structure remains intact after membrane formation. Additionally, the broad absorption band in the 1200–1000 cm^–1^ region arises from the overlap of the C–O–C stretching vibrations of PLA and the P–O–C stretching vibrations of D2EHPA, confirming the presence of both components in the membrane.^17,37^ The absence of new bands and the preservation of the main spectral features indicate that, similarly to the PVC-based system, the incorporation of D2EHPA into the PLA matrix occurs without covalent bond formation.^12,17,20^ Taken together, the FTIR results support the successful incorporation of D2EHPA into both PIM-1 and PIM-2 while preserving the chemical structure of the polymer matrices. The observed spectral differences between the PVC- and PLA-based membranes are consistent with differences in polymer–carrier interactions, suggesting that polymer selection may contribute to the physicochemical characteristics of the resulting membranes.^12,17^

#### 3.2.2 SEM/EDS Microscopy Measurements

Figure 5 presents the SEM micrographs and the corresponding EDS analyses of PIM-1 and PIM-2. The surface of PIM-1 exhibits a relatively homogeneous and compact morphology, with no evidence of macroscopic phase separation or pore formation at the magnification examined as shown in Figure 5b. This behavior is consistent with PVC-based polymer membranes prepared by solvent casting, in which the polymer matrix forms a dense and continuous phase capable of retaining low-molecular-weight additives within the membrane structure.^12,19^ In contrast, PIM-2 (Figure 5a) exhibits a rougher and porous surface with more pronounced textural features. This morphology can be associated with the semicrystalline nature of PLA and the development of microstructural heterogeneity during solvent evaporation, which is commonly observed in cast PLA films due to crystallization and chain rearrangement phenomena.^42,43^

**Figure 5:**
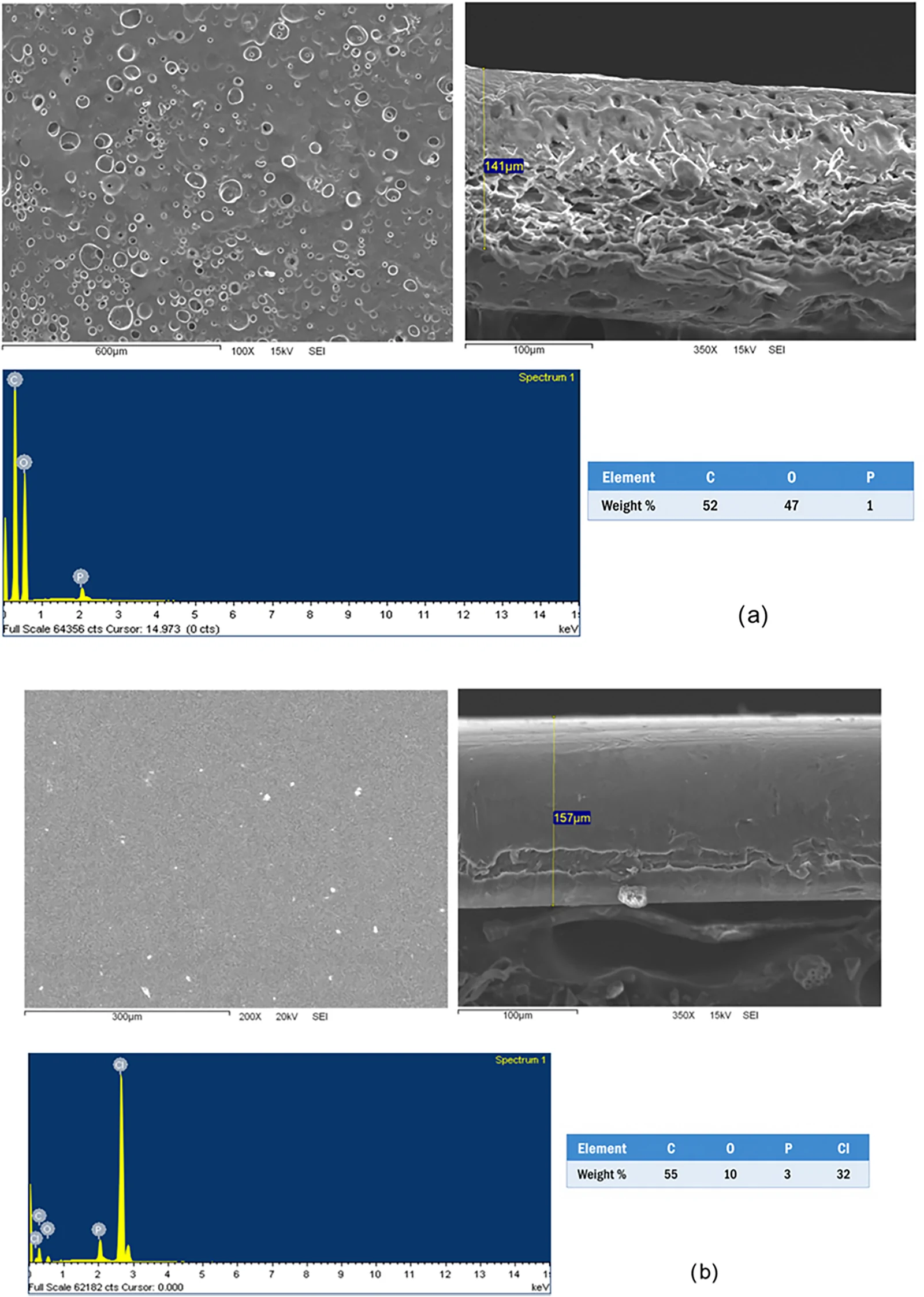
Scanning electron microscopy (SEM) images of polymer inclusion membranes (PIMs) prepared via solvent casting. Surface morphology of (a) PIM-2 and (b) PIM-1, along with their corresponding cross-sectional views respectively and energy-dispersive X-ray spectroscopy (EDS) elemental analyses are also presented.

Despite the increased roughness, no cracks or major defects are observed, indicating adequate membrane integrity. The absence of macroscopic defects is particularly important for polymer inclusion membranes, since structural discontinuities may facilitate carrier loss and reduce membrane reproducibility during prolonged operation.^19,20^ The EDS spectrum of PIMs confirms C and O as dominant elements, while the absence of Cl is consistent with the replacement of PVC by PLA.^12,19^ The presence of P provides direct evidence of the incorporation of the organophosphorus carrier (D2EHPA) in the membranes of the PLA and PVC matrix. When considered together with the FTIR-ATR band shifts discussed previously, the SEM/EDS observations are consistent with the formation of composite membranes stabilized by noncovalent polymer–carrier interactions, with hydrogen-bonding and dipole–dipole interactions being the most plausible interactions, which suppress macroscopic phase separation and favor carrier immobilization within the polymeric structure.^44,45^ Furthermore, the detection of phosphorus without evidence of elemental segregation is consistent with the incorporation of D2EHPA throughout the membrane matrix, without indications of macroscopic phase separation. Such a distribution is desirable because it can contribute to more uniform membrane properties and more reproducible transport behavior.

Cross-sectional SEM images allow direct observation of internal morphology and membrane thickness. PIM-1 exhibits a continuous cross-section with a well-defined thickness of approximately 157 μm, and no visible voids or delamination (Figure 5b). This compact internal structure is indicative of good compatibility between PVC and the embedded carrier, supporting the formation of structurally continuous membranes, as typically observed for plasticized or additive-containing PVC films.^19^ PIM-2 also shows a continuous cross-sectional structure (Figure 5a), although a slightly different internal texture is observed, consistent with differences in PLA chain packing and semicrystalline organization.

The PLA-based membrane presents a slightly lower thickness (approximately 141 μm), which may be associated with higher shrinkage during solvent evaporation and distinct densification behavior compared to amorphous polymers, as reported for solution-cast PLA systems.^42,43^ The SEM-based thickness measurements complement the mechanical characterization, since thickness is required for accurate stress calculations and can significantly influence deformation behavior. Therefore, cross-sectional SEM provides a key structural parameter for interpreting the tensile properties reported in the corresponding section, as commonly emphasized in polymer film and membrane mechanics studies.^35^ Based on these observations, the SEM and EDS analyses confirm the successful formation of both PIM-1 and PIM-2. The differences observed between the two membranes are mainly attributed to the intrinsic properties of the polymer matrices (PVC and PLA), which influence surface texture and membrane thickness. These observations are consistent with previous studies on polymer-based composite membranes, where polymer selection governs morphology, compatibility and long-term structural stability.^12,19,42,43^

#### 3.2.3 Thermal Analysis (TGA–DSC)

Thermogravimetric analysis (TGA) and differential scanning calorimetry (DSC) were carried out to evaluate the effect of incorporating the organophosphorus carrier D2EHPA into PIM-1 and PIM-2 matrices. The thermograms (Figure 6a,b) reveal degradation behaviors consistent with those reported for PVC- and PLA-based systems in the literature.

**Figure 6:**
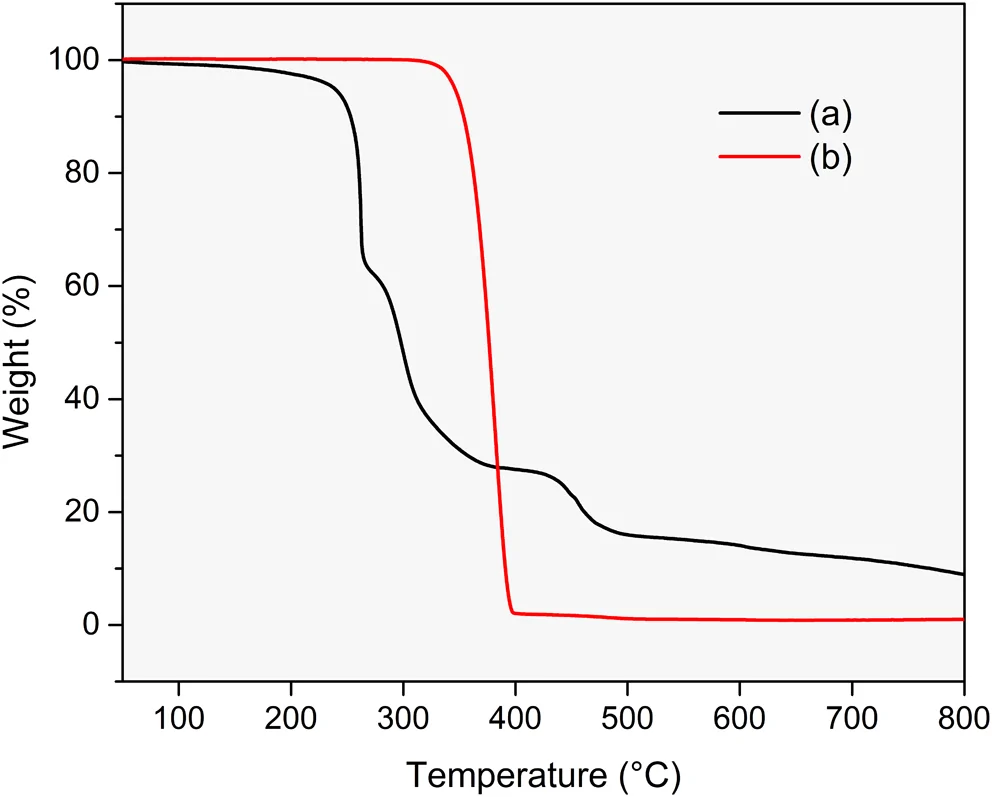
TGA curves of (a) PVC-based and (b) PLA-based polymer inclusion membranes.

The TGA curve of PIM-1 (Figure 6a) exhibits a multistep degradation pattern typical of PVC-based materials. A minor weight loss below 150 °C is observed, which can be attributed to the removal of adsorbed moisture and volatile species weakly retained in the membrane. The main degradation step occurs between approximately 250 and 380 °C. This behavior is characteristic of PVC thermal decomposition and is widely attributed to a two-stage mechanism: (i) dehydrochlorination leading to the formation of conjugated polyene sequences, followed by (ii) backbone scission and aromatization reactions.^22–24,46^ Neat PVC typically exhibits degradation onset temperatures between 270 and 300 °C.^24^ The slightly lower onset temperature observed for PIM-1 suggests that D2EHPA exerts a plasticizing effect, increasing polymer chain mobility and facilitating HCl elimination, as commonly reported for plasticized PVC systems.^21,24,47^ At temperatures above 400 °C, gradual mass loss is associated with the decomposition of the carbonaceous residue formed during earlier degradation stages, which is consistent with the formation of aromatic structures during PVC degradation.^22^

In contrast, PIM-2 (Figure 6b) shows a predominantly single-step degradation profile, typical of aliphatic polyesters such as PLA. The main mass loss occurs between approximately 280 and 360 °C and is attributed to random ester bond cleavage, intramolecular transesterification, and depolymerization mechanisms.^26,29^ Neat PLA generally exhibits degradation onset temperatures in the range of 300–330 °C.^26^ The slight shift toward lower temperatures observed for PIM-2 indicates that D2EHPA reduces intermolecular interactions within the PLA matrix. This behavior can be rationalized by hydrogen bonding interactions between the phosphoric acid group of D2EHPA and the ester carbonyl groups of PLA, which may disrupt chain packing and enhance segmental mobility. Similar plasticizing effects have been reported in polymer inclusion membranes containing carriers or ionic liquids.^31,48^ Unlike PVC, PLA does not typically generate significant char residue, resulting in a more defined and narrower degradation event.^26^

According to the obtained results, thermogravimetric analysis revealed that PIM-1 exhibited slightly higher thermal stability than PIM-2, with 5% mass loss occurring at approximately 220 and 210 °C, respectively. In both systems, the main degradation step occurred between 250 and 380 °C. The glass transition temperatures (*T*_g_), determined from DSC, are included in Table 3 for comparison with the thermal degradation parameters obtained from TGA. The *T*_g_ values were approximately 60 °C for PIM-1 and 58 °C for PIM-2. These values are in agreement with literature data, where neat PVC typically exhibits glass transition temperatures in the range of 75–85 °C and PLA shows *T*_g_ values between 55 and 65 °C.^21,27,28^ The slightly reduced *T*_g_ values observed in both PIMs support that D2EHPA acts as an internal plasticizer, decreasing intermolecular cohesion and increasing polymer chain mobility. The absence of additional thermal transitions further suggests good compatibility between D2EHPA and the polymer matrices, indicating homogeneous membrane formation without detectable phase separation by DSC.^12,20,31^

**Table 3:** Thermal Degradation Parameters (TGA) and Glass Transition Temperatures (*T*_g_) of the Prepared PIMs

membrane	temperature of 5% mass loss T_5_% (°C)	temperature of 10% mass loss T_10_% (°C)	temperature of 50% mass loss T_50_% (°C)	glass transition temperature *T*_g_ (°C)
PIM-1	220	245	360	60
PIM-2	210	235	345	58

In summary, thermal characterization demonstrates that while D2EHPA incorporation induces moderate plasticization, the dominant degradation mechanisms remain characteristic of each polymer matrix, supporting the suitability of these membranes for separation applications. The differences in thermal degradation behavior between PIM-1 and PIM-2 are primarily governed by the intrinsic structure of the polymer backbone. PVC undergoes multistep degradation dominated by dehydrochlorination and subsequent backbone scission, whereas PLA exhibits a more homogeneous single-step degradation associated with ester bond cleavage.^22,26^

Although the incorporation of D2EHPA slightly reduces the onset degradation temperature in both systems, the materials remain thermally stable well above typical operational conditions for membrane separation processes (generally below 100 °C). Therefore, the addition of the organophosphorus carrier does not significantly compromise the structural stability of the membranes.

#### 3.2.4 Contact Angle Measurements

PIM transport and stability properties are also determined by the membrane hydrophilic/hydrophobic balance. When a membrane is too hydrophilic, it will dissolve in water and leach out the carrier; when it is too hydrophobic, it is susceptible to biofouling and low extraction efficiency. The PIM must thus be hydrophobic enough to prevent the carrier from leaching out and hydrophilic enough to increase the amount of time the membrane surface is in contact with the feed solution.^4^

Figure 7 presents the average water contact angle (θ) values for the prepared PIMs as a function of the polymer matrix. The influence of the polymer support on the surface wettability of the membranes was evaluated through static contact angle measurements. The reported values correspond to the average of ten independent measurements performed on different regions of each membrane surface. As observed, the nature of the polymer matrix slightly modifies the surface behavior of the membranes. The PVC-based PIM exhibited an average contact angle of 59.2 ± 4°, whereas the PLA-based PIM showed a slightly higher value of 64.3 ± 6°. These contact angle values indicate moderately wettable surfaces, as values below 90° are generally associated with hydrophilic behavior.^49–51^ The increase observed for the PLA-based membrane suggests a modest change in apparent surface wettability associated with the polymer matrix composition. These results indicate that the polymer support plays a role in determining surface wettability, which may influence membrane–solute interactions and, consequently, transport performance.^20^

**Figure 7:**
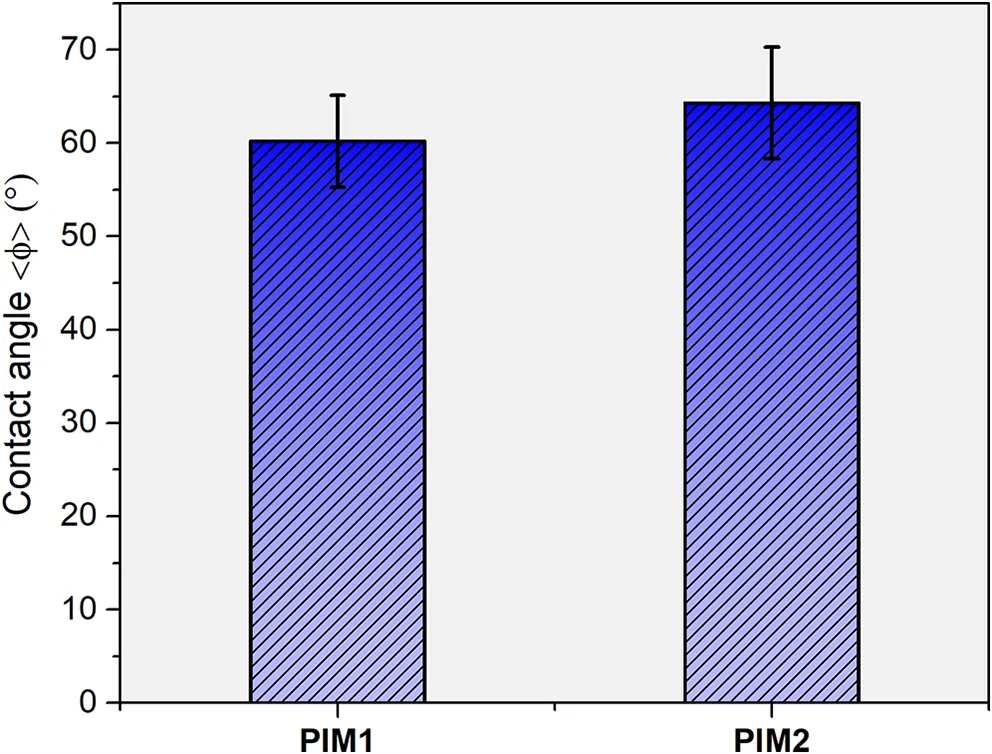
Variation of membrane surface contact angle with the different types of PIMs.

Similar contact angle values have been reported for PVC-based PIMs containing Aliquat 336, typically ranging between 55–75°, depending on plasticizer content.^3,4,20^ In contrast, PLA-based membranes often exhibit slightly higher surface energy due to the presence of ester groups, which may influence wettability and transport behavior.^26,43^ D2EHPA contains phosphoryl (P=O) and P–O groups capable of participating in dipole–dipole and hydrogen-bond-type interactions, which may contribute to the observed surface wettability.^17^ Collectively, these observations suggest that the measured contact angles reflect the combined influence of the polymer matrix and the incorporated carrier. From a functional standpoint, the moderate wettability exhibited by both membranes is relevant for aqueous separation processes, since surface wettability can influence interfacial resistance, mass transfer kinetics, fouling behavior, and membrane stability.^3,4^

Taken together, the contact angle results indicate that both PIM-1 and PIM-2 exhibit a balanced hydrophilic–hydrophobic character compatible with aqueous membrane applications. Although replacing PVC with PLA slightly increased the contact angle, the wettability remained within the range commonly reported for polymer inclusion membranes, suggesting that PLA preserves the surface characteristics required for efficient membrane operation in aqueous systems.

#### 3.2.5 Mechanical Analysis

The mechanical behavior of PIM-1 and PIM-2 membranes was evaluated from stress–deformation curves obtained using a texturometer (Figure 8). Both membranes display an initial region of low-stress deformation attributable to elastic response, followed by deviation from linearity as deformation progresses, consistent with the viscoelastic and plastic behavior typically reported for polymer membranes containing low-molecular-weight additives or plasticizing agents.^52^

**Figure 8:**
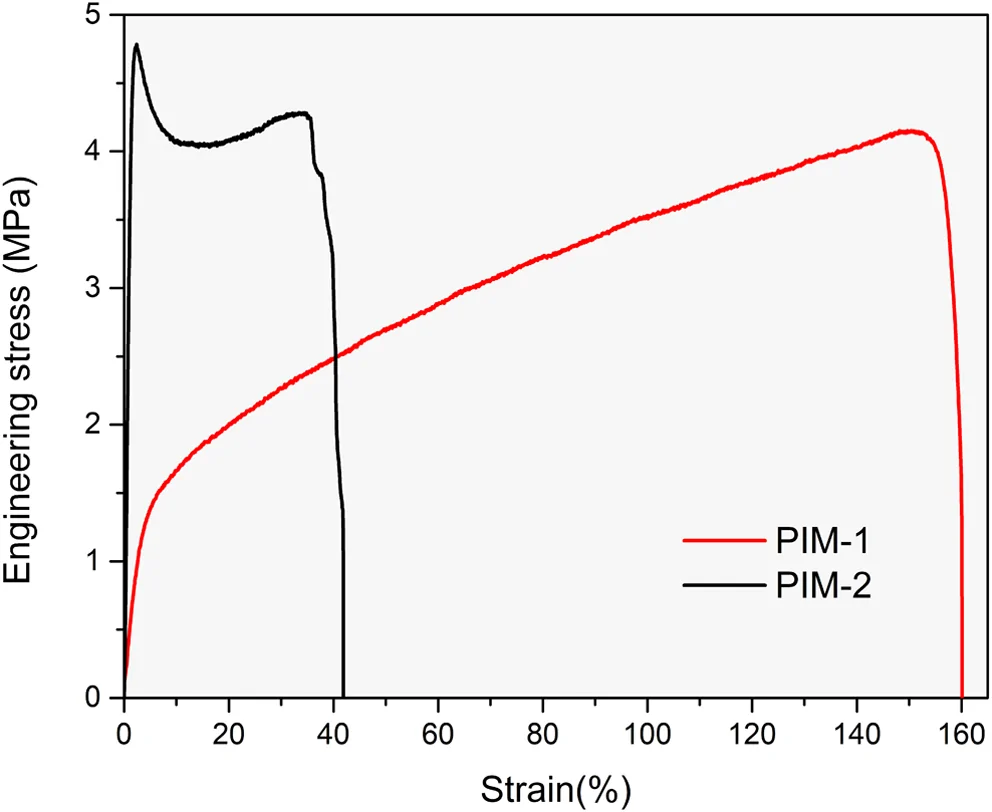
Stress–strain curves of the prepared polymer inclusion membranes obtained using a texture analyzer. Black curve corresponds to PIM-2 and red curve to PIM-1. The curves illustrate the mechanical behavior of the membranes under tensile deformation.

The mechanical behavior of the prepared membranes was evaluated from the stress–strain curves. Although the maximum stress values are relatively similar, PIM-2 reaches 4.28 MPa whereas PIM-1 attains 4.78 MPa. In contrast, a clear difference in deformation is observed, with PIM-1 showing a significantly higher elongation at break of 160.1% compared to 41.87% for PIM-2, corresponding to an approximately 3.8-fold increase. The apparent Young’s modulus was estimated from the slope of the initial quasi-linear region of the stress–strain curves for each specimen, and the reported values correspond to the mean ± standard deviation (*n* = 5). The PVC-based membrane exhibited a modulus of 31.8 ± 2.1 MPa, whereas the PLA-based membrane showed a significantly higher value of 369.1 ± 7.5 MPa. The nearly one-order-of-magnitude increase in Young’s modulus highlights the dominant influence of the polymer matrix on membrane stiffness, indicating that the mechanical response is governed primarily by the intrinsic characteristics of the supporting polymer rather than by the common carrier incorporated into both formulations.

These results reveal a marked contrast in mechanical response between both systems, where the lower modulus and higher elongation observed for PIM-1 indicate a more ductile and flexible material, while PIM-2 exhibits a significantly stiffer and less extensible profile. This behavior is consistent with the widely reported plasticization effect induced by low-molecular-weight additives, including organophosphorus compounds, when incorporated into polymer matrices, which enhance chain mobility and elongation at break while potentially reducing stiffness depending on miscibility and matrix rigidity.^21,52^ The observed differences can be primarily attributed to the nature of the polymer support, as also reported for polymer inclusion membranes and related systems.^21,42,52^ PVC-based systems typically exhibit greater flexibility due to weaker intermolecular interactions and increased chain mobility, particularly in the presence of plasticizing agents, whereas PLA is widely recognized as a more rigid and brittle polymer, often associated with semicrystalline organization and stronger intermolecular interactions that restrict chain mobility and reduce elongation at break.^21,43,52^ Therefore, although D2EHPA contributes to plasticization in both systems, the PLA matrix limits extensibility, resulting in a stiffer membrane with lower ductility compared to the PVC-based PIM. This finding demonstrates that the plasticizing effect of D2EHPA is strongly dependent on the polymer matrix, which ultimately determines the balance between flexibility and rigidity in the resulting membranes.^21,39,52^

From an application perspective, these results suggest that PIM-1 may offer improved tolerance to handling and mechanical deformation due to its higher strain at failure, whereas PIM-2 exhibited greater stiffness and dimensional stability, which may be advantageous in applications where preservation of membrane geometry is essential for maintaining reproducible transport properties. Examples include flat-sheet polymer inclusion membrane modules used for the continuous extraction or transport of metal ions and organic compounds, passive sampling devices deployed for environmental monitoring, and analytical preconcentration systems, where membrane deformation may alter the effective diffusion path length and the active transport area. In these configurations, higher dimensional stability can contribute to more reproducible transport performance during prolonged operation. Nevertheless, the greater rigidity of PLA-based membranes should be balanced against their lower elongation at break, depending on the intended application.^3,4,7^ These results demonstrate that polymer matrix selection provides an effective strategy for tailoring the mechanical performance of polymer inclusion membranes according to the requirements of their intended application.^3,4,19^

## 4 Conclusions

Polymer inclusion membranes based on PVC and PLA containing D2EHPA were successfully prepared by solvent casting and comparatively characterized to evaluate the influence of the polymer matrix on their physicochemical properties. FTIR confirmed the successful incorporation of D2EHPA while preserving the characteristic chemical structures of both polymer matrices, whereas SEM/EDS revealed continuous membranes with distinct morphologies and no evidence of macroscopic phase separation. Thermal analysis demonstrated matrix-dependent degradation behavior and thermal stability, while water contact angle measurements indicated moderately wettable surfaces for both membranes. The most pronounced differences were observed in the mechanical properties, with PVC-based membranes exhibiting greater ductility and lower stiffness, whereas PLA-based membranes showed substantially higher stiffness and lower elongation at break. Overall, these findings demonstrate that polymer matrix selection governs the physicochemical and mechanical behavior of D2EHPA-based polymer inclusion membranes, with PVC providing greater flexibility and PLA representing a promising biodegradable alternative for applications requiring enhanced stiffness and dimensional stability. Although the observed changes in membrane properties are consistent with increased chain mobility following D2EHPA incorporation, the specific contribution of its plasticizing effect cannot be conclusively established from the present results. Therefore, further studies evaluating transport performance, long-term stability, and different carrier-to-polymer ratios are needed to better elucidate the role of D2EHPA and optimize membrane design for separation applications.
